# Medium development and production of carotenoids and exopolysaccharides by the extremophile *Rhodothermus marinus* DSM16675 in glucose-based defined media

**DOI:** 10.1186/s12934-022-01946-7

**Published:** 2022-10-23

**Authors:** Israt Jahan Mukti, Roya R. R. Sardari, Thordis Kristjansdottir, Gudmundur O. Hreggvidsson, Eva Nordberg Karlsson

**Affiliations:** 1 Division of Biotechnology, Department of Chemistry, Lund University, Naturvetarvägen 14, 22100 Lund, Sweden; 2 Matis Ohf, Vinlandsleid 12, 113 Reykjavik, Iceland; 3 Department of Biology, School of Engineering and Natural Sciences, University of Iceland, Sturlugata 7, 102 Reykjavik, Iceland

**Keywords:** Thermophile, *Rhodothermus marinus* DSM 16675, Exopolysaccharides, Carotenoids, Defined medium, Medium development

## Abstract

**Background:**

The marine thermophilic bacterium *Rhodothermus marinus* can degrade many polysaccharides which makes it interesting as a future cell factory. Progress using this bacterium has, however, been hampered by limited knowledge on media and conditions for biomass production, often resulting in low cell yields and low productivity, highlighting the need to develop conditions that allow studies of the microbe on molecular level. This study presents development of defined conditions that support growth, combined with evaluation of production of carotenoids and exopolysaccharides (EPSs) by *R. marinus* strain DSM 16675.

**Results:**

Two defined media were initially prepared: one including a low addition of yeast extract (modified Wolfe’s medium) and one based on specific components (defined medium base, DMB) to which two amino acids (N and Q), were added. Cultivation trials of *R. marinus* DSM 16675 in shake flasks, resulted in maximum cell densities (OD_620 nm_) of 2.36 ± 0.057, cell dry weight (CDW) 1.2 ± 0.14 mg/L, total carotenoids 0.59 × 10^–3^ mg/L, and EPSs 1.72 ± 0.03 mg/L using 2 g/L glucose in DMB. In Wolfe’s medium (supplemented by 0.05 g/L yeast extract and 2.5 g/L glucose), maximum OD_620 nm_ was 2.07 ± 0.05, CDW 1.05 ± 0.07 mg/L, total carotenoids 0.39 × 10^–3^ mg/L, and EPSs 1.74 ± 0.2 mg/L. Growth trials at 5 g/L glucose in these media either failed or resulted in incomplete substrate utilization. To improve reproducibility and increase substrate utilization, a screening of macroelements (e.g. phosphate) in DMB, was combined with use of trace elements and vitamins of the modified Wolfe’s medium. The resulting defined minimal *R. marinus* medium, (DRM), allowed reproducible cultivations to a final OD_620nm_ of 6.6 ± 0.05, CDW 2.85 ± 0.07 mg/L, a maximum specific growth rate (µ_max_) of 0.26 h^−1^, total carotenoids 0.77 × 10^–3^ mg/L and EPSs 3.4 ± 0.17 mg/L in cultivations supplemented with up to 5 g/L glucose.

**Conclusion:**

A minimal defined medium (DRM) was designed that resulted in reproducible growth and an almost doubled formation of both total carotenoids and EPSs. Such defined conditions, are necessary for systematic studies of metabolic pathways, to determine the specific requirements for growth and fully characterize metabolite production.

**Supplementary Information:**

The online version contains supplementary material available at 10.1186/s12934-022-01946-7.

## Background

Extremophiles, including thermophiles, have been offered as sources of stable and active enzymes at elevated temperature. Despite the interest in extremophiles for this purpose, only a limited number of model organisms for growth and metabolite production, have been reported [1]. The chemoorganotrophic aerobe *Rhodothermus marinus* has been attracting interest due to its thermostable enzymes and its ability to produce carotenoids and exopolysaccharides (EPSs) [2, 3], and the genome sequence of strain DSM 4252^T^ is available [4]. The type strain of *R. marinus* was first isolated from a submarine (2–3 m depth) hot spring in Isafjardardjup, NW Iceland [5]. Cultivation parameters of strain DSM 4252^T^ have however been difficult to follow due to aggregation of the cells, and genetic tools have not been developed for this strain due to the presence of restriction enzymes [6]. Another native strain, *R. marinus* DSM 16675 (also known as *R. marinus* PRI 493, MAT 493 and ISCAR-493), also isolated from an Icelandic biotope, has more frequently been used for research due to its lower degree of cell aggregation and its deficiency in restriction enzymes, which allowed introduction of genetic materials and subsequent strain development [3, 7].

*R. marinus* DSM 16675 is able to grow in several complex media such as Lysogeny broth (LB), marine broth, and the ATCC medium 1598 or 1599 [8]. These media have been used for pre-cultures of *R. marinus* and to produce carotenoids and EPSs, which are two major metabolites of this microorganism. However, in order to study the metabolic pathways and to determine the specific requirements for growth and metabolites production of *R. marinus* DSM 16675, a chemically defined medium is needed [9]. The aim of this study was to optimize a defined minimal medium for *R. marinus* DSM 16675 in order to allow studies of the growth kinetics, achieve higher cell density, and monitor metabolites formation using a sole carbon source.

## Results

### Inoculum preparation of *R. marinus* DSM 16675 using glucose as the sole carbon source

After reviving the − 80 °C glycerol stocks of strain DSM16675 on ATCC 1599-agar plates, a two-step inoculum preparation procedure in liquid medium was used, in which the inocula were produced in baffled shake flasks using 2 g/L glucose. The cells were transferred from the agar plates to the desired liquid medium, grown to the exponential growth phase and then transferred to a fresh medium, in order to increase the inoculum size and obtain high levels of viable cells in a short time. Inoculum preparation is often an overlooked step, that can have an impact on the outcome of the cultivation process e.g. in terms of productivity [10]. In this work, transfer in the early exponential phase was important for cell viability (data not shown), and the repeated liquid culture inoculation procedure was important to allow use of an inoculum from a liquid medium of identical composition as the medium used in the cultivation.

### Growth of *R. marinus* DSM 16675 in two defined media: defined medium base (DMB), and modified Wolfe’s medium

A modification of the ATCC medium 1599 (replacing the Tryptone and Yeast extract with the amino acids N and Q) [11], here termed: defined medium base (DMB), and a modified Wolfe’s medium with low amounts of yeast extract [12] (Table 1) have, with some variations, been used to cultivate *R. marinus* DSM 4252^T^ in the past.

**Table 1:** Macroelements and additives in the DMB [11] and the modified Wolfe’s media [12, 13]

Ingredients (DMB)	Amount (per liter in DMB)	Ingredients (modified Wolfe’s)	Amount (per liter in modified Wolfe’s)
Nitrilotriacetic acid	2.01 g	Tris	4.85 g
CaSO_4_.2H_2_O	0.4 g	CaSO_4_.2H_2_O	0.04 g
MgCl_2_.6H_2_O	2 g	MgCl_2_.6H_2_O	0.20 g
NaCl	10.0 g	NaCl	10.0 g
KH_2_PO_4_	0.27 g	KH_2_PO_4_	0.30 g
Na_2_HPO_4_	0.28 g	K_2_HPO_4_	0.50 g
NH_4_Cl	0.5 g	NH_4_Cl	0.5 g
Ferric citrate (C_6_H_5_FeO_7_)	0.06 g		
Trace elements solution DMB (Table 4)	5 mL	Wolfe’s trace element solution (Table 4)	5 mL
Vitamins solution DMB (Table 5)	1 mL	Wolfe’s vitamins solution (Table 5)	5 mL
Asparagine	0.002 g	Yeast extract	0.05 g
Glutamine	0.002 g		
Distilled water	Up to 1 L	Distilled water	Up to 1 L
Adjust the pH to 7.2 by NaOH (6 M)		Adjust the pH to 7.2 by NaOH (6 M)	

The cell densities obtained using these media have however been low, and cell proliferation has not always been obtained, indicating some type of deficiency. Moreover, the modified Wolfe’s medium, contained small amounts of yeast extract, which is a drawback when a defined medium is needed. These two available types of media nevertheless provided a starting point for screening. To obtain cultivation data, DMB and modified Wolfe’s medium were used in shake flask cultivations supplemented with glucose as carbon source at two levels: 2 and 5 g/L for DMB medium and 2.5 and 5 g/L glucose for modified Wolfe’s medium, respectively. It should be noted that there is no growth without supplementing a carbon source and glucose was used as the main source of carbon in these cultivations. Without addition of glucose, the amino acids N and Q in low concentrations, or low supplementation of yeast extract in DMB and Wolfe’s medium, respectively, are the only available carbon sources. Consequently, no or very limited growth could be observed (no measurable growth curve) without glucose, as carbon limitation was reached very early.

**Fig. 1:**
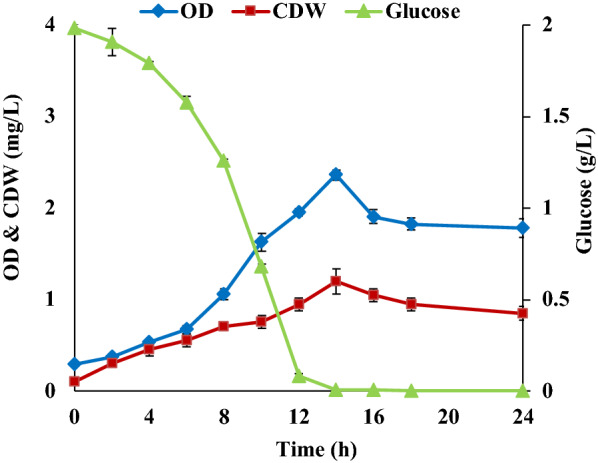
Growth of *R. marinus* DSM 16675 in DMB supplemented with 2 g/L glucose. The data in the figure is given as an average ± std of two experiments

Cultivation in DMB medium (Table 1) with 2 g/L glucose resulted in repeatable growth, a maximum cell density (OD_620 nm_) of 2.36 ± 0.057 (1.2 ± 0.14 mg/L CDW) after 14 h cultivation, a maximum specific growth rate (µ_max_) of 0.22 h^−1^ and complete consumption of glucose (Fig. 1). Despite repeated inoculations, cultivations in DMB medium with 5 g/L glucose gave inconsistent results, and it was difficult to start cell proliferation.

**Fig. 2:**
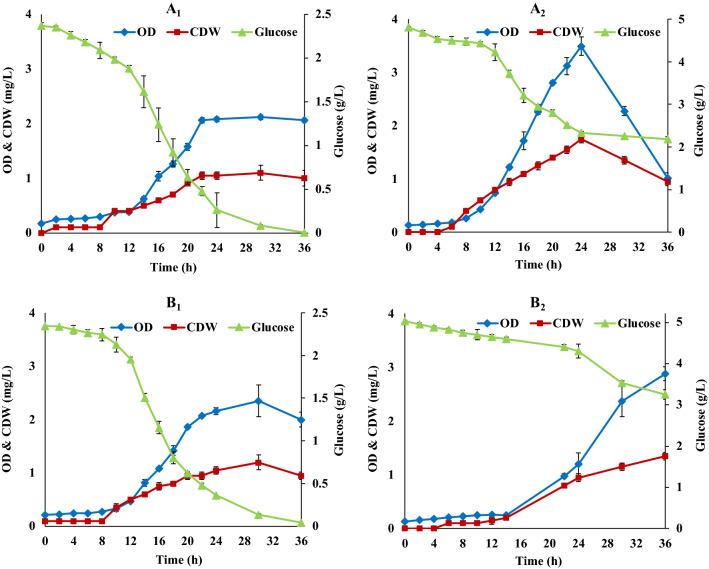
Growth of *R. marinus* DSM 16675 in modified Wolfe’s medium with 0.05 g/L yeast extract (**A**_**1**_, **A**_**2**_) and without yeast extract (**B**_**1**_, **B**_**2**_) supplemented with 2.5 (**A**_**1**_, **B**_**1**_) and 5 g/L (**A**_**2**_, **B**_**2**_) glucose as substrate. The data in the figure is given as an average ± std of two experiments

In parallel, shake flask cultivations of *R. marinus* DSM 16675 were carried out in modified Wolfe’s medium (Table 1) with and without yeast extract (0.05 g/L) and at 2.5 or 5 g/L glucose supplementation (Fig. 2). The results showed a similar specific growth rate when yeast extract supplementation was used (μ_max_ of 0.24 h^−1^, Fig. 2A1, A2), while it decreased to a μ_max_ of 0.17 h^−1^ and 0.14 h^−1^ respectively, in the cultivations without this supplementation (Fig. 2B1, B2).

The maximum cell density was not affected significantly by the yeast extract addition, resulting in final OD_620 nm_ of 2.07 ± 0.05 (1.05 ± 0.07 mg/L CDW) after 22 h with yeast extract (Fig. 2A1) and 2.35 ± 0.29 (1.2 ± 0.14 mg/L CDW) after 30 h (Fig. 2B1) without yeast extract. In both cases 2.5 g/L glucose supplementation was used. With 5 g/L glucose, the cell density obtained was 3.5 ± 0.17 (1.75 ± 0.07 mg/L CDW) after 24 h (Fig. 2A2) with yeast extract, and 2.88 ± 0.13 (1.35 ± 0.07 mg/L CDW) after 36 h without yeast extract (Fig. 2B2). Overall, the final cell mass did not respond to the increased addition of carbon source, showing limitations of other nutrients and also substrate inhibition at high concentration, as the glucose consumption was incomplete after 36 h.

### Product formation in DMB and modified Wolfe’s medium

**Fig. 3:**
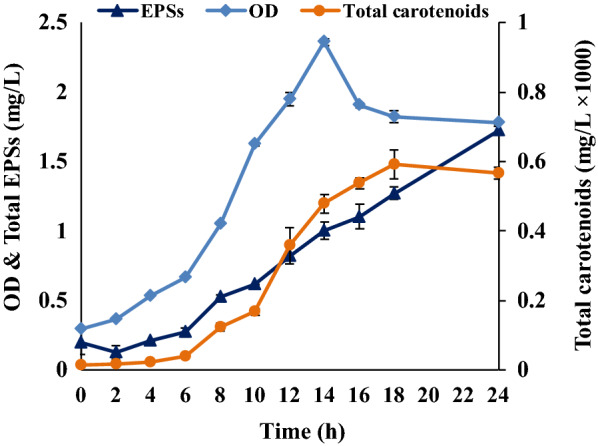
Production of carotenoids and EPSs by *R. marinus* DSM 16675 in DMB supplemented with 2 g/L glucose. The data in the figure is given as an average ± std of two experiments

**Fig. 4:**
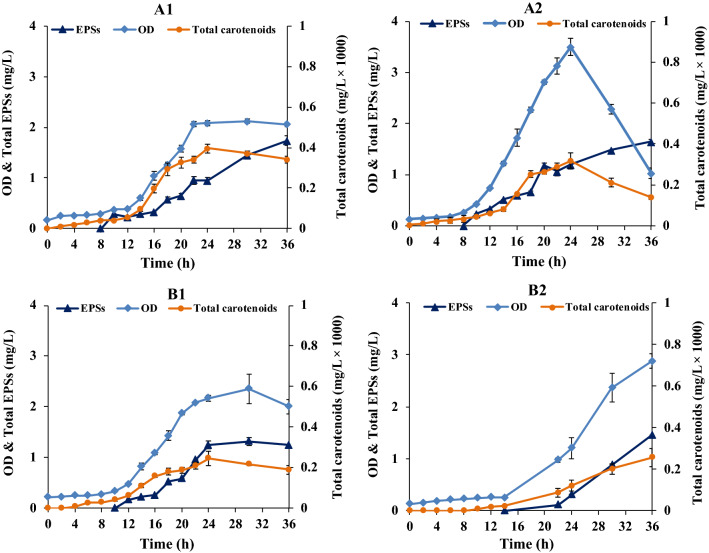
Production of carotenoids and EPSs by *R. marinus* DSM 16675 in modified Wolfe’s medium with 0.05 g/L yeast extract (**A**_**1**_, **A**_**2**_) and without yeast extract (**B**_**1**_, **B**_**2**_) supplemented with 2.5 (**A**_**1**_, **B**_**1**_) and 5 g/L (**A**_**2**_, **B**_**2**_) glucose as substrate. The data in the figure is given as an average ± std of two experiments

The formation of carotenoids and EPSs, two products of potential interest, produced by *R. marinus* DSM 16675 were also evaluated using the respective medium (Figs. 3 and 4).

#### Carotenoid production

Cultivation in the DMB medium with 2 g/L glucose resulted in the highest total carotenoid production, with an estimated concentration of 0.59 × 10^–3^ mg/L, at the end of the growth phase after 18 h cultivation (Fig. 3). The corresponding production of carotenoids in modified Wolfe’s medium with and without yeast extract (0.05 g/L) and with supplementation of 2.5 and 5 g/L glucose, are shown in (Fig. 4). In this medium, production of carotenoids was lower, despite reaching a similar cell density. The highest carotenoids production (estimated to 0.39 × 10^–3^ mg/L and 0.24 × 10^–3^ mg/L) was reached in the modified Wolfe’s medium with 2.5 g/L glucose with and without yeast extract, respectively, after 24 h cultivation (Fig. 4A1, B1). Increased glucose supplementation did not result in increased carotenoid production (Fig. 4).

#### EPSs production

The results for growth and EPSs production of *R. marinus* DSM 16675 in DMB and modified Wolfe’s media are shown in Table 2. EPSs production generally peaked late in the respective cultivation. Highest EPSs production in DMB was in the stationary phase, after 24 h (Fig. 3). The EPSs production in the modified Wolfe’s medium with yeast extract supplementation (Fig. 4) was in the same range as the EPSs monitored for DMB, but highest EPSs production occurred later in the cultivation (after 36 h). Also, it was shown that a higher glucose concentration (5 g/L) did generally not boost the production, resulting in lower yield and productivity. Moreover, production was slightly decreased when yeast extract was excluded from the cultivation.

**Table 2:** Growth and EPSs production of *R. marinus* DSM 16675 in DMB and modified Wolfe’s media

Medium	Initial glucose (g/L)	Cultivation time (h)	OD	CDW (mg/L)	Consumed glucose (g/L)	Produced EPSs (mg/L)	Yield (mg EPSs/g glucose)	Productivity (mg/L/h)
DMB	2	24	1.78 ± 0.09	0.85 ± 0.07	2	1.72 ± 0.03	0.75 ± 0.01	0.071 ± 0.001
Modified Wolfe’s medium with yeast extract	2.5	36	2.06 ± 0.04	0.95 ± 0.07	2.42	1.74 ± 0.20	0.63 ± 0.21	0.040 ± 0.01
	5	36	1.02 ± 0.09	0.75 ± 0.07	2.82	1.65 ± 0.19	0.63 ± 0.07	0.045 ± 0.005
Modified Wolfe’s medium without yeast extract	2.5	30	2.35 ± 0.29	1.2 ± 0.14	2.37	1.32 ± 0.09	0.57 ± 0.04	0.043 ± 0.004
	5	36	2.88 ± 0.13	1.15 ± 0.07	1.76	1.45 ± 0.1	0.82 ± 0.01	0.040 ± 0.002

Growth of *R. marinus* DSM 16675 in the modified Wolfe’s medium supplemented with 5 g/L glucose without yeast extract showed a longer lag phase, and also resulted in a lower final cell density and less consumed glucose (1.77 g/L) compared to the medium containing yeast extract (2.82 g/L consumed glucose) during 36 h of cultivation. It seems, however, that glucose was consumed primarily for production of EPSs, rather than for cell growth which can explain the corresponding yield and productivity in the medium supplemented with 5 g/L glucose without yeast extract.

Overall, the carotenoid production was higher in the DMB medium compared to the modified Wolfe’s medium. The yield and productivity of EPSs in DMB with 2 g/L glucose was slightly higher than in the Wolfe’s medium. Due to these facts, the DMB medium was chosen for a screening design, aiming at increasing glucose consumption and growth at higher initial glucose concentration, allowing higher cell density and/or production of the two targeted metabolites.

### Three step medium design combined with cultivation experiments

#### Evaluation of the influence of macroelement concentrations on glucose consumption

To adapt the DMB medium for growth at higher concentration of the carbon source, a one factor at a time screening strategy [14] was applied, using 5 g/L glucose. In the first step, the concentration of four macro-elements (CaSO_4_, MgCl_2_, PO_4_^3−^, and NH_4_Cl) were set at two levels (Table 3). Only one component was set at a higher level at a time while keeping the others at the concentrations in the original DMB medium (Table 1).

**Table 3:** Variables and levels used in the modified OFAT screening strategy

Variables	Low level (DMB) final concentration	High level final concentration
CaSO_4_ .2H_2_O	0.4 g/L	2 g/L
MgCl_2_ .6H_2_O	2 g/L	4 g/L
Phosphate stock solution (1) (5.44 g/L KH_2_PO_4_ and 5.67 g/L Na_2_HPO_4_)	0.27 g/L of KH_2_PO_4_ and 0.28 g/L of Na_2_HPO_4_ (50 mL of phosphate stock solution (1)/L)	–
Phosphate stock solution (2) (27.22 g/L KH_2_PO_4_ and 28.39 g/L Na_2_HPO_4_)	–	1.36 g/L of KH_2_PO_4_ and 1.41 g/L of Na_2_HPO_4_ (50 mL of phosphate stock solution (2)/L)
NH_4_Cl (53.49 g/L and 160.4 g/L stock solutions)	0.535 g/L (10 mL of 53.49 g/L NH_4_Cl stock solution/L)	1.6 g/L (10 mL of 160.4 g/L NH_4_Cl stock solution/L)

**Fig. 5:**
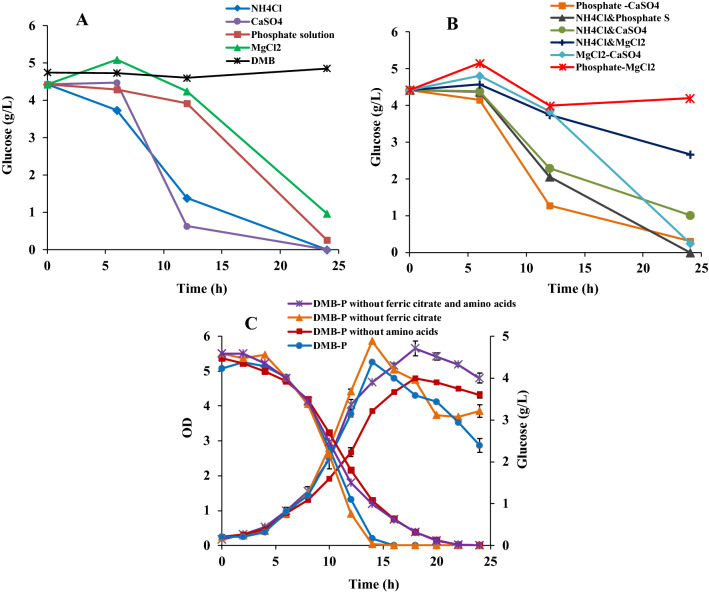
**A** One factor at a time screening analysing glucose consumption by *R. marinus* DSM 16675 in DMB medium containing 5 g/L glucose and higher level of one microelement in a single set of experiments. **B** Consumption of 5 g/L glucose by *R. marinus* DSM 16675 in DMB containing high level of two variables in a single set of experiments. **C** The growth profile of *R. marinus* DSM 16675 and glucose consumption. The data is given as an average ± std of two experiments

Five experiment sets were designed, including the original DMB medium (Table 1) as a control, which was used in the three step inoculum preparation with the initial glucose concentration of 2 g/L and in the cultivation experiment (as control) with initial glucose concentration of 5 g/L. In all cases the glucose concentration in the medium was determined at different time points in each cultivation (Fig. 5A).

As seen in (Fig. 5A), all four experiments resulted in growth and glucose consumption, which was not the case in the original DMB medium. The consumption of glucose in the medium containing either a high level of NH_4_Cl or high level of CaSO_4,_ was faster than at the other two conditions (high phosphate or high MgCl_2_). Assuming a linear approximation in certain intervals, high level NH_4_Cl resulted in a consumption rate of 0.11 g/L/h during the first 6 h and reached a glucose level of 1.4 g/L after 12 h (with the rate 0.4 g/L/h in the 6 h − 12 h interval) and glucose was completely consumed after 24 h. High level of CaSO_4_ resulted in a 6 h lag-phase, but after that glucose was consumed at a high rate (approximated to 0.64 g/L/h), reached 0.63 g/L after 12 h and was completely consumed after 24 h.

Glucose consumption was slower using a high level of phosphate or MgCl_2_, but both cultivations had similar trends in the substrate consumption rate, which began after 12 h of cultivation and reached 0.26 g/L with the rate of 0.3 g/L/h (high phosphate) and 0.97 g/L with the rate of 0.27 g/L/h (high MgCl_2_), respectively. The cells did not grow in the control medium (DMB at 5 g/L), indicating that a higher level of at least one of the macro-elements is needed to allow growth at increased concentration of the carbon source. CaSO_4_ and NH_4_Cl had the strongest effects on glucose utilization, but the high levels did in both cases cause precipitation in the medium, complicating measurements of cell density. Hence, phosphate or MgCl_2_ were considered to be more beneficial components to modify.

Six parallel experiments were also performed using high levels of two components at a time (Additional file 1: Table S1) and keeping others at the concentration of the original DMB medium in order to screen the effect of two factor combinations on *R. marinus* DSM 16675 glucose consumption (Fig. 5B).

The Fig. 5B showed that combination of high level of phosphate solution with high level of CaSO_4_ and NH_4_Cl had similar effect on glucose consumption as when using them at high level, separately (Fig. 5A). Also, the combination of high level of MgCl_2_ with the other variables in high level had negative impact on glucose consumption by *R. marinus* DSM 16675. These results indicated that the growth of *R. marinus* DSM 16675 was not improved by combination of two variables at high level compared to using them at high level, separately. The reason might be due to increased precipitation due to the combination of two compounds at high concentration (instead of only one). Based on these results and in order to select components that minimized the risk of precipitation in the medium, DMB with a high level of phosphate (DMB-P) was chosen for further screening.

#### Evaluation of the importance of ferric citrate and amino acids on growth and glucose consumption

In the second step of the screening, the effect of the presence/absence of ferric citrate (12.24 g/L) and the two amino acids N and Q (1% w/v, respectively) in DMB-P with 5 g/L glucose was evaluated in four sets of *R. marinus* DSM 16675 cultivation experiment: (i) with both amino acids and ferric citrate (control) (ii) without the amino acids, (iii) without ferric citrate and (iv) without both amino acids and ferric citrate (Fig. 5C).

As seen in (Fig. 5C), the four cultivations resulted in very similar growth and glucose consumption curves, but with somewhat faster glucose consumption rate in presence of the amino acids. The maximum rate of glucose consumption of *R. marinus* DSM 16675 with and without ferric citrate, separately, was 0.43 g/L/h and 0.48 g/L/h, with a final cell density (OD_620 nm_) of 5.26 and 5.86, respectively. Without amino acids, the glucose consumption was slightly lower (0.36 g/L/h, and 0.39 g/L/h without both amino acids and ferric citrate) while the final cell density varied more (4.75 without amino acids, and 5.65 ± 0.21 without both amino acids and ferric citrate). The simplest version (DMB-P_min_), without ferric citrate and the amino acids N and Q, was selected for further design of the minimal medium as the variations were relatively small when included.

#### The importance of trace elements and vitamins solutions

The developed medium (DMB-P_min_) showed improvement over the original DMB. However, repeated trials using the developed medium (DMB-P_min_), showed that the repeatability was poor. Eleven cultivations were performed with three steps inoculum preparation (1 step on agar plate and 2 steps in liquid medium) and the qualitative repeatability (meaning the % of inoculations that resulted in cell proliferation) of the 1st step of inoculum preparation (in liquid medium) was 73%, the qualitative repeatability of the 2nd inoculation step was 62.5% (of those successfully proliferated in the 1st step), and for the shake flask cultivation step growth was obtained in 40% of the cultures inoculated from the 2nd step. This indicated that there may be a deficiency of elements needed at very low concentrations, e.g. trace elements or vitamins, after repeated cultivations. The trace element and vitamin solutions of the modified Wolfe’s medium contain more compounds than the corresponding DMB medium solutions. Therefore, the trace element and vitamin solutions originally used in the DMB-P_min_ medium were replaced with those of the Wolfe’s solutions (Tables 4 and 5).

**Table 4:** Ingredients in the DMB [11] and Wolfe’s [12, 13] trace-element solutions

Ingredients (DMB trace)	Amount (per liter DMB-trace)	Ingredients (modified Wolfe’s trace)	Amount (per liter modified Wolfe’s trace)
Nitrilotriacetic acid	12.8 g	Nitrilotriacetic acid	1.5 g
		MgSO_4_.7H_2_O	3.0 g
		NaCl	1.0 g
FeCl_2_ .4H_2_O	1 g	FeSO_4_·7H_2_O	0.1 g
MnCl_2_ .4H_2_O	0.5 g	MnSO_4_·H_2_O	0.5 g
CoCl_2_ .6H_2_O	0.3 g	CoCl_2_·6H_2_O	0.1 g
		CaCl_2_	0.1 g
CuCl_4_ .2H_2_O	0.05 g	ZnSO_4_·7H_2_O CuSO_4_·5H_2_O AlK(SO_4_)_2_·12H_2_O	0.1 g 0.01 g 0.01 g
Na_2_MoO_4_ .2H_2_O	0.05 g	Na_2_MoO_4_·2H_2_O	0.01 g
H_3_BO_3_	0.02 g	H_3_BO_3_	0.01 g
NiCl_2_ .6H_2_O	0.02 g		
Distilled water	Up to 1 L	Distilled water	Up to 1 L
Adjusting the pH to 7 by NaOH (6 M)		Adjusting the pH to 7 by KOH (2 M)

**Table 5:** Ingredients in the DMB [11] and Wolfe’s [12, 13] vitamins solutions

Ingredients vit-DMB	Amount (per liter in vit-DMB)	Ingredients (modified Wolfe’s vit)	Amount (per liter modified Wolfe’s vit)
		Thioctic acid	5 mg
		Folic acid	2 mg
		Riboflavin	5 mg
Thiamine	4 mg	Thiamine hydrochloride	5 mg
Nicotinic acid	4 mg	Nicotinic acid	5 mg
Biotin	4 mg	Biotin	2 mg
Ca-pantothenate	4 mg	Ca-pantothenate	5 mg
Cyanocobalamine	4 mg	Vitamin B_12_ (cobalamine)	0.1 mg
*p*-Aminobenzoic acid	4 mg	*p*-Aminobenzoic acid	5 mg
Pyridoxine	4 mg	Pyridoxine hydrochloride	10 mg
Distilled water	Up to 1 L	Distilled water	Up to 1 L

**Fig. 6:**
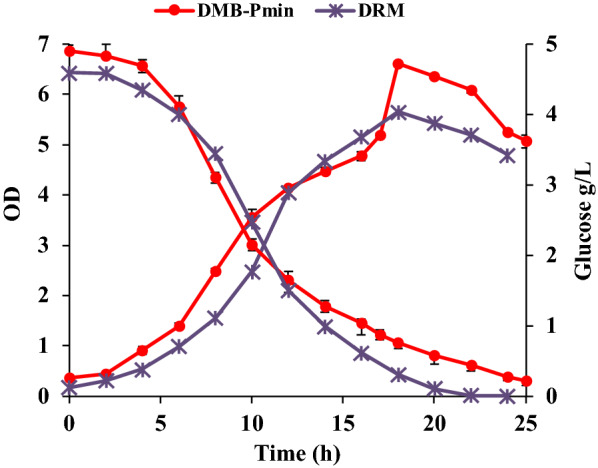
The growth profile of *R. marinus* DSM 16675 and glucose consumption

As seen in (Fig. 6), replacement of trace elements and vitamins solutions in the medium (now termed DRM (Defined *Rhodothermus* Medium) resulted in a small increase in final cell density (OD_620nm_ = 6.6 ± 0.05), while the maximum specific growth rate (μ_max_) was 0.26 h^−1^, which is in the same range as observed for both DMB (0.22 h^−1^) and modified Wolfe’s medium with yeast extract supplementation (0.24 h^−1^), and the same as in DMB-P_min_. The glucose consumption profile was also similar in DMB-P_min_ and DRM (Fig. 6). Repeated cultivation trials, using DRM were successful, allowing cell proliferation after repeated inoculations in the same medium (data not shown).

Consumption of glucose by *R. marinus* DSM 16675 in DRM medium started immediately with the rate of 0.05 g/L/h during the first 4 h of cultivation, and then increased significantly to the rate of 0.43 g/L/h for 8 h and then decreased to 0.12 g/L/h until the end of cultivation (Fig. 6). The apparent growth rate was not uniform (decreased after 12 h, but then increased again) which indicates either production of metabolites by the cells (reducing cell division) or aggregation. The apparent *R. marinus* DSM 16675 growth rate increased again after 17 h of cultivation after which the optical density decreased. This might be due to aggregation or cell lysis, that could be caused by either EPSs production or lack of some nutrients such as NH_4_Cl (nitrogen source) since there was still glucose in the medium. The component, NH_4_Cl, was not added to the medium at high level due to precipitation problem but can be added gradually during a fed-batch cultivation mode.

In DMB-P_min_, glucose consumption started after 2 h with the rate of 0.14 g/L/h and then after 6 h of cultivation increased significantly with the rate of 0.4 g/L/h for 8 h and then decreased to 0.14 g/L/h till the end of cultivation. The maximum consumption rate of glucose in both media was in the same range.

The possibility of *R. marinus* DSM 16675 to grow in DRM supplemented with higher concentration of glucose than 5 g/L, was also tested (10, 15 and 20 g glucose/L). *R. marinus* DSM 16675 grew in all cultures which means that there was no inhibitory effect of high concentration of sugars on cell growth (Additional file 1: Table S2). The maximum cell density (OD_620 nm_) and overall glucose consumption were, however, similar in all the cultivations which might be due to lack of other nutrients that can be added in a fed-batch mode.

#### Product formation comparison and correlation between growth and product formation in the original DMB and developed DRM media

**Fig. 7:**
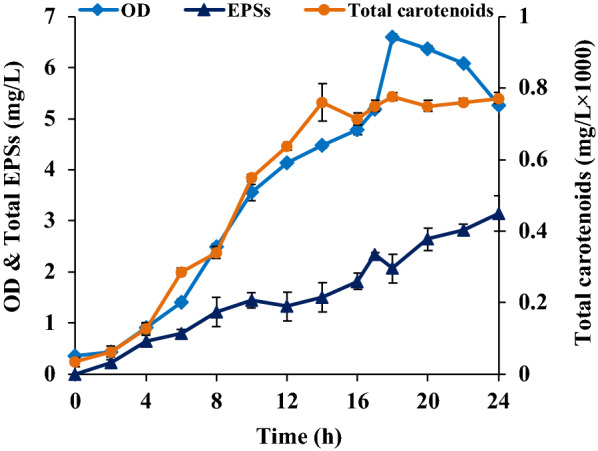
Production of carotenoids and EPSs by *R. marinus* DSM 16675 in DRM medium supplemented with 5 g/L glucose

The total carotenoids and EPSs produced by *R. marinus* DSM16675 in the DRM medium are shown in (Fig. 7). The highest total carotenoids concentration (0.77 × 10^–3^ mg/L) produced by *R. marinus* DSM 16675 was obtained in the DRM medium supplemented with 5 g/L glucose at 18 h cultivation.

EPSs production in DRM was also higher than in the other defined media, with the highest EPSs production (3.4 ± 0.17 mg/L) found after 24 h with the yield of 0.7 ± 0.04 mg EPSs/g consumed glucose and a volumetric productivity of 0.13 ± 0.006 mg/L/h. Both the carotenoid- and EPSs production were higher in DRM than in the DMB medium that was used as the starting point for the development.

Regression analysis between the CDW of *R. marinus* DSM 16675 and the produced metabolites in the DMB-medium at 2 g/L glucose and modified Wolfe’s medium containing yeast extract supplemented with 5 g/L glucose, showed moderate correlation between production of carotenoids and EPSs with increasing CDW (Table 6) while in modified Wolfe’s medium with and without yeast extract supplemented with 2.5 g/L glucose and without yeast extract supplemented with 5 g/L, showed strong correlation between production of and EPSs with increasing CDW (Table 6).

The CDW of the *R. marinus* DSM 16675 cultivated in DRM medium supplemented with 5 g/L glucose strongly correlated with the carotenoids concentration while the EPSs production was less well correlated. This is not surprising, as it is a consequence of EPSs production continuing into the stationary phase.

The final concentration of macro-elements and additives in the DRM and the preparation conditions are shown in Additional file 1: Table S3, respectively.

**Table 6:** Analysis of variance (ANOVA) for determination of the significance of CDW to the metabolites

Medium	Initial glucose (g/L)	r_Carotenoids_	*p*-Value_Carotenoids_	rEPSs	*p*-Value_EPSs_
DMB	2	0.8	< 0.01	0.7	< 0.05
Modified Wolfe’s medium with yeast extract	2.5	0.95	< 0.001	0.95	< 0.001
	5	0.96	< 0.001	0.83	< 0.01
Modified Wolfe’s medium without yeast extract	2.5	0.96	< 0.001	0.9	< 0.001
	5	0.97	< 0.001	0.92	< 0.001
DRM	5	0.95	< 0.001	0.76	< 0.01

(r) represented the correlation coefficient between CDW and metabolites

### Characterization of the purified produced EPSs by *R. marinus* DSM 16675 cultivated in DRM

The monosaccharide composition analysis of the purified EPSs (Additional file 1: Table S4) revealed that they were heteropolysaccharides with xylose in highest content followed by mannose, and arabinose. Two unidentified peaks were observed in all chromatograms (Additional file 1: Fig. S1). In comparison to the results obtained by previous cultivation of *R. marinus* DSM 16675 in marine broth supplemented with glucose [2], the ratio of xylose and mannose showed an increased molar ratio and galactose also appeared in the composition of produced EPSs in DRM.

### Functional group analysis

Functional group analysis of *R. marinus* DSM 16675 grown in DRM was done by FT-IR spectroscopy (Additional file 1: Fig. S2). The spectrum was similar to the FT-IR spectrum of EPSs produced by *R. marinus* DSM 16675 grown in marine broth and showed the same functional groups as previously identified [2]. Briefly, the IR spectra exhibited a broad peak at around 3303 cm^−1^ (range 3600–3200 cm^−1^) for O–H stretching vibration of the polysaccharide. The peak at 1635 cm^−1^ corresponded to a C=O stretching vibration of the *N*-acetyl group or protonated carboxylic. Also, at 1540 cm^−1^ a peak was observed which was assigned to the N–H deformation vibration of an amine group. The peak at 1521 was assigned to the secondary amid group. Another peak at 1412 cm^−1^ could be attributed to the symetric stretching of the COO^−^ group. The peak at 1219 cm^−1^ was corresponded to an O–S–O group that is an evidence of sulfate esters. The strong absorption at 1050 cm^−1^ in the range of 1200–1000 cm^−1^ which is anomeric region, was attributed to C–O–C and C–O groups in polysaccharides and suggested that a monosaccharide in the EPSs had a pyranose ring. The peak at 910 cm^−1^ had strong absorption which corresponds to a ß-glycosidic bond.

## Discussion

Development of a defined minimal medium for growth of *R. marinus* DSM 16675 is important for studies of metabolic pathways in the microorganism, to control consumption of the carbon source, and to introduce changes during process development. *R. marinus* can utilize several mono-(glucose, galactose) [5], di-(lactose, maltose, sucrose) [2] and polysaccharides (xylan) [15] as sole carbon source. The results of previous cultivation studies of *R. marinus* DSM 16675 on complex media supplemented with glucose as carbon source have shown that glucose may not be the most preferred carbon source for *R. marinus* DSM 16675 [8], and may also not be the most favored substrate for EPSs production [16]. One reason for this might be due to the thermal lability of glucose at high temperature as seen in the growth profile of the hyperthermophile *Pyrococcus furiosus* [17]. However, as glucose is the most established carbon source for analysis of metabolic routes, and often the first choice in defined media carbon source supplementation, it was chosen as the sole carbon source during the development of a defined minimal medium for *R. marinus* DSM 16675. In the first inoculum preparation from the agar plate to the liquid defined medium supplemented with 2 g/L glucose, it took a long time to reach the final cell density, OD_620 nm_ = 2. The second and third inoculum preparations resulted in cell proliferation in shorter time, indicating adaptation to the conditions.

The existing defined medium, DMB, was optimized by changing the concentration of one of the four medium components at a time: CaSO_4_, MgCl_2_, phosphate (KH_2_PO_4_ and Na_2_HPO_4_) or NH_4_Cl. The subsequent growth rates and glucose uptake rates were determined and an increased concentration of any of these components resulted in complete consumption of glucose (5 g/L) and better growth compared to the original medium. A genome-scale metabolic model of *R. marinus* [18] was used to estimate the required concentration of the main medium components for optimized growth and to facilitate interpretation of the results obtained here. The model was used to predict the growth rates, using default model constraints, and the resulting fluxes of the three metabolites: phosphate, sulfate and ammonium were compared to the in vivo data obtained here. The in vivo fluxes were approximated from Fig. 7, by calculating the complete consumption of the available metabolites in the medium by the obtained cell mass over the time that the cells were in growth phase (Additional file 1: Table S5). This analysis indicated that the original DMB medium contained barely enough phosphate to obtain maximum growth, which supports the decision to increase the phosphate concentration in the DRM medium. The analysis also indicated that the ammonium concentration in the DMB medium was too low, which supported the in vivo experiments where glucose was taken up faster with a higher ammonium concentration in the medium. Also, Dudman et al. [19] described that the nitrogen source increased the growth of *Rhizobium meliloti*, an EPSs producing bacterium, in defined medium, but the DMB medium with increased NH_4_Cl resulted in precipitation problems. This was most likely caused by the combined concentrations of NH_4_Cl, MgCl_2,_ and phosphate that have the tendency to make a struvite complex (MgNH_4_PO_4_·6H_2_O) in aqueous solutions [20]. For this reason, the ammonium concentration was not increased in the DRM medium.

The sulfate concentration used in the DMB medium was estimated to be sufficient, according to model simulations. However, in vivo growth of *R. marinus* in DMB medium with 10 times less sulfate did result in poor growth (data not shown, as growth experiments using different concentrations of sulfate were performed in a different lab with other minor medium changes). The calculated sulfate flux based on the model, indicated that the 10 times lower sulfate concentration was indeed too low to obtain maximum growth, strengthening the reliability of the model simulations. The highest glucose uptake rate in vivo was obtained using a higher concentration of CaSO_4_. It is known that Ca^2+^, Mg^2+^, and K^+^ ions are necessary for utilization of the substrate and production of polysaccharides as cofactors [21–23]. However, the solubility of calcium salts in aqueous solutions is low and higher concentration of CaSO_4_ caused precipitation of the salt in the medium, making a higher amount difficult to include in soluble form. In conclusion, the medium with higher levels of phosphate solution (DMB-P) was chosen for the batch cultivation studies displayed here since no precipitation was observed in the medium and the glucose was almost completely consumed after 24 h of *R. marinus* DSM 16675 cultivation. Further supplementation based on the simulations can be considered for feeding during cultivations in stirred tanks.

In the second step of medium screening, the added amino acids (N and Q) and ferric citrate were removed from the medium. The utilization rate of the glucose as carbon source by *R. marinus* DSM 16675 in DMB-P without amino acids was lower than that in the corresponding medium containing amino acids. On the other hand, the results indicated that *R. marinus* DSM 16675 growth was not strictly dependent on presence of the two amino acids. Moreover, a medium without the two amino acids might be beneficial for industrial use in future, as it will result in less expensive components. Hence, depending on the application, amino acids can be added or removed from the medium.

Iron is, in general, essential for bacteria due to its application in cellular respiration and DNA synthesis. In contradiction to this, *R. marinus* DSM 16675 grew better in the DMB-P without ferric citrate. The absence of this micronutrient might, however, be a reason for the lack of repeatability of the DMB-P_min_, as growth failed after several re-inoculations in the medium. Use of this element at high concentration might be toxic or inhibitory, and has been reported to have a negative effect on cell growth [24, 25]. Bacteria, in general, can take up ferrous iron (Fe^2+^) via a ferrous ion internalization system [26]. Therefore, ferric iron (Fe^3+^) should be converted to Fe^2+^ to simplify the uptake. This could be possible by *R. marinus* ferredoxin (GenBank: AF515798.1) [27] or ferric reductase (GenBank: ACY47438.1) [4]. High concentration of Fe^2+^ is, however, still toxic for the cells due to its tendency to react with oxygen to generate reactive oxygen species (ROS) which subsequently, can damage the proteins, DNA, and membrane lipids [25]. The concentration of iron as ferric citrate in DMB was 0.06 g/L and in the DMB trace elements solution, the concentration of ferrous iron chloride was 1 g/L. Removal of ferric iron citrate and replacement the DMB trace elements solution by the Wolfe’s trace elements solution might have increased the bioavailability of iron for the *R. marinus* DSM 16675 to uptake at a low concentration [28], as iron was added as Fe^2+^ at a very low concentration (FeSO_4_·7H_2_O, 0.1 g/L) in Wolfe’s trace elements solution (Table 4). Another possibility is that *R. marinus* might utilize innate immunity [29], restricting iron to very low concentrations, preventing its uptake [30].

Wolfe’s trace elements solution and vitamins solution was evaluated to investigate cell proliferation when repeatedly using the resulting final medium (termed DRM). Wolfe’s trace elements solution contains Mg^2+^, SO_4_^2+^, Ca^2+^, K^+^, and NaCl which were not present in the DMB trace element solutions, but as these components were supplied as medium ingredients, they might not be necessary. The Zn^2+^ is a cofactor for many enzymes and may be a valuable addition to the trace elements solution (Table 4), making only small amounts necessary.

The Wolfe’s vitamins solution (Table 5) contains all the vitamins present in the DMB vitamins solutions, but also include folic acid, riboflavin, and thioctic acid. Moreover, a higher amount of the vitamin solution (5×) is used. Folic acid, a B-vitamin, is necessary for production of some amino acids [31] and cannot be replaced by any other vitamins or chemicals. The p-aminobenzoic acid is necessary for biosynthesis of folic acid [32]. The concentration of p-aminobenzoic acid in Wolfe’s vitamins solutions (5 mg/L) was higher than that of the DMB vitamins solutions (4 mg/L), and additional folic acid (2 mg/L) was added to the Wolfe’s vitamins solution, which may be advantageous for growth in a minimal medium. Riboflavin is a precursor of the coenzymes flavin mononucleotide (FMN) and flavin adenine dinucleotide (FAD), which are involved in oxidative metabolisms in most bacteria [33]. It can be a replacement of iron as enzyme cofactor flavin-dependent enzymes, due to iron restriction as described above and subsequently, downregulation of the non-essential iron-dependent enzymes (iron-sparing) aiming at keeping iron for fundamental functions [28]. Also, thioctic acid (lipoic acid) was added to the vitamin solution since it is an essential cofactor for oxidative metabolism [34, 35] in aerobic bacteria. Based on this, the change of vitamin solution, may be an important factor for the increased repeatability of the *R. marinus* cultivations.

Bacterial secondary metabolites are non-essential products where the yield mainly depends on the cultivation niche and medium ingredients [36]. The bioactive carotenoids can have a protective role against radiation, oxidation, desiccation and high temperature by reinforcing the mechanical strength, flexibility, rigidity to the cell membrane and lowering the susceptibility to lipid peroxidation [37–40]. The experiments conducted were considering the optimum conditions for *R. marinus* DSM 16675 without relating to the external light source, indicating that the total carotenoids production was related to the cell biomass production. The positive correlation of total carotenoids production with CDW in the media used here, supports this hypothesis in the present study (Table 6).

The yield of EPSs by the *R. marinus* DSM 16675 was higher in the modified Wolfe’s medium, than when yeast extract was supplied to the same, but longer lag phase was seen, and glucose consumption level was lower (Table 2, Figs. 6 and 7) indicating an imbalanced nutritional status. The consumption of external carbon source (glucose) by the *R. marinus* DSM 16675 in DRM, however, had a positive influence on both total carotenoids and EPSs production (Fig. 7), but may have resulted in conditions that stressed the bacterial cells.

*Rhodothermus marinus* DSM 16675 has the capability to produce a variety of carbohydrate degrading enzymes allowing utilization of different mono, di, oligo, and polysaccharides including its own EPSs, when carbon availability is low. Hence, EPSs productivity may vary dependent on nutrient availability. The apparent EPSs production started at the exponential phase in DRM with 5 g/L glucose supplementation. However, it cannot be excluded that the reason might be competition between cell growth and EPSs production, and better availability of medium components for EPSs production at the stationary phase may be beneficial [41].

## Conclusion

In the present study, a defined minimal medium (DRM) was developed using glucose as sole carbon source in shake flask cultivation for *R. marinus* DSM 16675.

The total carotenoids production and EPSs productivity and yield was higher in the DRM than in the previous defined media used for growth of *R. marinus*. This study unambiguously created the opportunity to portray the possibilities of the bacterium for different purposes, including evaluation of production of carotenoids and EPSs, at defined conditions. Different external carbon sources can now be studied in a more controlled environment (i.e. bioreactor) allowing analysis of production pathways.

## Methods

All the materials and reagents were analytical grade and purchased from Sigma-Aldrich unless otherwise specified. Yeast extract and tryptone were obtained from Difco and sodium hydroxide (NaOH, 50%) from Merck.

### Bacterial strains and inoculum preparation

*Rhodothermus marinus* DSM 16675 was received as freeze-dried samples from Matís ohf, Reykjavík, Iceland and revived on ATCC agar 1599 plate (*Thermus* enhanced medium with 1% NaCl) [42] for 24–48 h at 65 °C (Ecotron, Infors) and a half loopful (10 µL loop) culture were re-cultured in a 250 mL baffled shake flask containing 25 mL liquid medium (Lysogeny broth (LB) including 10 g/L tryptone, 10 g/L NaCl, and 5 g/L yeast extract, and incubated at 65 °C and 200 rpm until the optical density at 620 nm (OD_620 nm_) reached more than 2 (approximately 18–20 h). Then, 5 mL of the resulting culture (10% v/v as inoculum volume) was transferred into 45 mL of LB in a 500 mL baffled shake flask and incubated at same temperature and shaking until the OD_620 nm_ reached more than 2 (approximately 8–10 h). This culture was used as the stock culture by adding 0.5 mL of the culture to 0.5 mL of sterile glycerol solution (50%) in a cryotube and kept in − 80 °C freezer.

For inoculum preparation, the cells from the stock culture were grown on an ATCC 1599 agar plate (first step of inoculum preparation) and then transferred into a 250 mL baffled shake flask containing the variant of the medium used in the cultivation to be investigated with different concentration of glucose (2, and 5 g/L) as a sole carbon source, and incubated at 65 °C and 200 rpm until the OD_620 nm_ reached between 2 to 4 (second step of inoculum preparation, approximately 18 h). Then, 5 mL of the resulting culture (10% v/v as inoculum volume) was transferred into a 500 mL baffled shake flask containing 45 mL of desired medium and incubated at 65 °C and 200 rpm until the OD_620 nm_ reached between 2 and 4 (third step of inoculum preparation, approximately 12 h). This culture was used as inoculum (10% v/v) for shake flask cultivation.

### Cultivation medium preparation

#### Preparation of the two cultivation media, a defined medium base (DMB) and modified Wolfe’s medium

Based on the components used in the medium used by Blucher et al. [11] and the salts of ATCC1599, a defined medium base (DMB) was made (Table 1). The DMB was prepared with 20 mL of filter-sterilized l-glutamine (Gln, 1% w/v) and 20 mL of filter-sterilized l-asparagine (Asn, 1% w/v). The concentration of nitriloacetic acid was increased to 2 g to avoid of small precipitation that happened by adding of 1.32 g (the original value) to the medium solution. The aqueous glucose stock solution (100 g/L) was prepared and filter-sterilized. All the filter-sterilized stock solutions were sterilized using 0.2 µm sterile polypropylene syringe filters. Every time freshly prepared medium was made, a final glucose concentration of 2 or 5 g/L was used, and the final pH was adjusted to 7.2 using 6 M NaOH.

The modified Wolfe’s medium [12, 13] was prepared as described in (Table 1). The modified Wolfe’s medium was prepared without and with yeast extract (0.05 g/L), separately. All the filter-sterilized stock solutions were sterilized using 0.2 µm sterile polypropylene syringe filters. Freshly prepared medium was used in all cultivations, with a final glucose concentration of 2.5 or 5 g/L and the final pH was adjusted to 7.2 using 6 M NaOH.

### Cultivation conditions

The *R. marinus* DSM 16675 cultivations were performed at 65 °C with constant shaking of 200 rpm and 10% v/v inoculum using either DMB (with or without supplementation with the amino acids N and Q) or modified Wolfe’s medium with and without yeast extract supplementation. The samples were taken every 2 h for further analysis. The glucose concentration in all media at the 2nd and 3rd step of inoculum preparation was 2 g/L except for the final optimized medium in which cells could manage to grow at 5 g/L glucose.

### Three step screening of the effect of medium component concentrations by a one factor at a time strategy

DMB was used as medium to screen the influence of substrate consumption or cell growth by varying the concentration of macroelement medium components using a modified one-factor-at-a-time screening strategy [14].

In the first step of screening, four factors from the medium macroelements were selected, and screened in cultivations of *R. marinus* DSM 16675 (Table 3). Two sets of experiments were performed. In one set of experiments one of the selected components (Table 3) was set at high level keeping the other selected components at low level. The remaining medium components were added as described for DMB (Table 1). Glucose concentration was 2 g/L in the medium for preparation of inoculum at 2nd and 3rd step of inoculum preparation, and 5 g/L in all cultivation experiments. The original DMB medium (Table 1) was used as control. In another set of experiments, 6 parallel experiments were performed using high levels of two components at a time, keeping other selected components at low level, and the other medium components at the original level. The cultivations were performed as single experiments at 65 °C with constant shaking of 200 rpm and 10% v/v inoculum using 50 mL falcon tubes containing 25 mL of culture medium. Samples were taken after 0, 6, 12, and 24 h of cultivation for further analysis.

In the second step of screening, four sets of experiments were performed, again using DMB with the glucose concentration of 2 g/L for the inoculum preparation. The culture medium was the optimum medium chosen from the first screening step (DMB-P), which was used as control in this step. The other three cultivations contained the same medium without (a) ferric citrate, (b) without amino acids (l-glutamine and l-asparagine), and (c) without both ferric citrate and amino acids. The cultivations were performed in duplicate at 65 °C with constant shaking of 200 rpm and 10% v/v inoculum in 1 L baffled shake flasks containing 100 mL of culture medium. Samples were taken every 2 h of cultivation for further analysis. This, resulted in additional modification (DMB-P_min_).

In the third step, the trace elements solution and vitamins solution were simply replaced by the Wolfe’s trace elements and Wolfe’s vitamins solution (Tables 4 and 5) [12, 13] in the DMB-P_min_ (lacking ferric citrate and the amino acids N and Q). This medium was termed DRM (Defined *Rhodothermus* medium). Cultivation of *R. marinus* DSM 16675 was carried out in duplicate in 1 L baffled shake flask containing 100 mL of DRM, in presence of 5 g/L glucose. The cultivation was performed at 65 °C with constant shaking of 200 rpm and 10% v/v inoculum (prepared in similar medium with the glucose concentration of 5 g/L) samples were taken every 2 h of cultivation for further analysis.

### Analytical methods

#### Optical density and cell dry weight

One milliliter of sample was used for cell growth measurement as optical density at 620 nm (OD_620 nm_) and the quantitative cell growth was evaluated based on cell dry weight (CDW). To determine CDW, 1 mL of sample was centrifuged for 3 min at 16,000×*g* (Sigma PK) to separate the cells from the medium. Then, the pelleted cells were washed with ultrapure water and centrifuged, and the water was discarded. After that, 2 mL of 0.05 M EDTA sodium salt solution was added to the cells and the sample was shaken in a rocking table for 4 h at 4 °C. Then the cell pellets were harvested by centrifugation and washed two times with ultrapure water. After discarding the water by centrifugation, 0.5 mL ultrapure water was added to the cells and then the cells were transferred to pre-heated and weighed aluminum boats and dried in an oven at 105 °C. The cell dry weight was measured periodically until a stable dry weight was observed.

#### Carotenoid production

The carotenoids were extracted from the *R. marinus* DSM 16675 by using a method described by Biehler et al. [43]. One milliliter of sample was pelleted at 16,000×*g* for 3 min and re-suspended in 1 mL of acetone (99.5%, Merck, Germany). After incubation for 1 h with constant shaking on a rocking table at room temperature, the cells were separated by centrifugation at 16,000×*g* for 3 min and then the absorbance of the supernatant was measured by a UV quartz cuvette (Hellma) in a UV/Vis spectrophotometer (Pharmacia biotech, Ultrospec 1000) at 450 nm. Acetone as lysis solution was used as control.

The major carotenoid in *R. marinus* DSM 16675 is salinixanthin as described by Ron et al. [3]. Therefore, the concentration of the total carotenoids from *R. marinus* DSM 16675 can be estimated using the Beer–Lambert equation and then plotted versus cultivation time:

1 $$C = {\text{A}} \times 969/\left( {\upvarepsilon _{\uplambda } \times {\text{d}}} \right)$$

where C is the concentration of the carotenoid (g/L), A is the measured absorbance, ɛ is the extinction coefficient at specific wavelength [L/(mol × cm)], and d is the width of the cuvette (usually 1 cm). The molecular weight of salinixanthin is 969 (g/mol) and the extinction coefficient of salinixanthin is predicted to be in the range of 125,000–140,000 [L/(mol × cm)] range [44]. In this study the mean value [132,000 L/(mol × cm)] was used as the extinction coefficient for determination of the carotenoid concentration.

#### Substrate consumption and carbohydrate analysis

Substrate (glucose) consumption and monosaccharides analysis (for determination of total produced EPSs) were performed as described by Ron et al. [8]. The samples were analyzed with high performance anion exchange chromatography with pulsed amperometric detection (HPAEC-PAD, Thermo Fisher Scientific, Waltham, USA) using Dionex CarboPac PA-20 guard column coupled Dionex CarboPac PA-20 analytical column. As eluents ultrapure water, 2 mM NaOH, and 200 mM NaOH were used with three separate pumps. Separation occurred at 23 min of running time by 62.5% ultrapure water and 37.5% of 2 mM NaOH mixture with an isocratic flow of 0.5 mL/min. The column was reinforced each time with 200 mM NaOH for 2 min at 0.5 mL/min flow rate. ED40 electro-chemical detector was used to detect the glucose consumption and monosaccharide content of EPSs.

Determination of glucose concentration as a remaining substrate in the culture medium was done by filtering the cell-free culture medium through a 0.2 μm polypropylene filter into a plastic vial after proper dilution and analysis by HPAEC-PAD.

The EPSs were extracted and analyzed by a method described by Sardari et al. [2]*.* One milliliter of sample was pelleted down using centrifuge at 13,000 rpm for 3 min. After adding the fourfold volume of ethanol (99.5%) to the cell-free supernatant, the crude EPSs was precipitated by keeping the samples at 4 °C for 1–2 days. Then, the precipitation was collected by centrifuging at 3890×*g* for 10 min at 4 °C (Sigma 316PK), air dried in a fume hood to evaporate the remaining ethanol and lyophilized (Labconco freeze dry system). The obtained crude EPSs from different cultures were hydrolyzed, by treating with sulfuric acid (72% w/w) for 30 min and then adding ultrapure water to the final concentration of (4% w/w) and then heated at 100 °C for 3 h in a heating block. Later, the solution was neutralized with 0.1 M Ba(OH)_2_·H_2_O followed by centrifugation at 3980×*g* for 10 min. After collecting the supernatant the monosaccharide content of the crude EPSs was analyzed with HPAEC-PAD. The cultivation medium was used as control.

Purification of the crude EPSs was done by size exclusion chromatography using a HPLC system (Agilent Technologies, USA) equipped with Refractive-Index detector (ERC-7510, ERMA INC) and Sephacryl S-200 HR (1 × 103–8 × 104 Da) (HiPrep, GE healthcare life sciences) column (16 mm × 600 mm). Ultrapure water was used as an eluent at the flow rate of 0.3 mL/min for 500 min.

The yield (Y) and volumetric productivity of the produced EPSs (VP) by *R. marinus* DSM 16675 in each shake flask cultivation were calculated as follows:

2 $$Y = {{\left[ {final\;EPSs - initial\;EPSs } \right]} \mathord{\left/ {\vphantom {{\left[ {final\;EPSs - initial\;EPSs } \right]} {\left[ {final\;glu\cos e {-}initial\;glu\cos e} \right]}}} \right. \kern-\nulldelimiterspace} {\left[ {final\;glu\cos e {-}initial\;glu\cos e} \right]}}$$

3 $$VP = {{\left[ {final\;EPSs - initial\;EPSs} \right]} \mathord{\left/ {\vphantom {{\left[ {final\;EPSs - initial\;EPSs} \right]} {t_{{{\text{total}}}} }}} \right. \kern-\nulldelimiterspace} {t_{{{\text{total}}}} }}$$

The t_*total*_ indicated the total time (h) of the shake flask cultivation, and $\left[ {EPSs} \right]$ is the EPSs formation during cultivation.

Glucose consumption rate was calculated as follows:

4 $$rglucose = \Delta glucose/\Delta t$$

The r indicated for the rate of glucose consumption at the certain intervals.

The regression analysis was performed by data analysis tool from Excel software to evaluate the relation between the cell growth and produced metabolites (EPSs and Carotenoids).

## Supplementary Information

**Additional file 1: Table S1.** Screening experiments of four macromolecules (CaSO_4_, MgCl_2_, PO_4_^3−^, and NH_4_Cl) with one factor at a time screening strategy (A, B, C, D, E) and combination of high levels of two factors at a time and keeping others at the concentrations in the original DMB medium (BC, BD, BE, CD, CE, DE). **Table S2.** The growth profile of *R. marinus* DSM 16675 in DRM medium with different concentration of glucose as sole carbon source. **Table S3.** The preparation condition of DRM. (A) Preparation condition for stock solutions (B) The preparation condition for DRM with final concentration in the medium. The pH is adjusted to 7.2 by NaOH (6 M) after addition of the remaining water. **Table S4.** The relative monosaccharide composition (molar ratio) of the purified EPSs produced by *R. marinus* DSM 16675 in RDM. The amount of glucose is set as 1. **Table S5.** Uptake and secretion rates of selected metabolites. In vivo rates were determined from experimental data in Fig. 7. In silico rates were obtained by simulating maximum growth rate in a genome-scale metabolic model of *R. marinus*. **Figure S1.** The monosaccharides chromatograms of the purified EPSs produced by *R. marinus* DSM 16675 in DRM with two unidentified peaks. **Figure S2.** FT-RI spectrum of the purified exopolysaccharide from *R. marinus* DSM 16675 grown in DRM.

## Data Availability

All dataset generated for this study are included in the article and Additional file.
