# Correction: Medium development and production of carotenoids and exopolysaccharides by the extremophile *Rhodothermus marinus* DSM16675 in glucose-based defined media

**DOI:** 10.1186/s12934-023-02048-8

**Published:** 2023-03-13

**Authors:** Israt Jahan Mukti, Roya R. R. Sardari, Thordis Kristjansdottir, Gudmundur O. Hreggvidsson, Eva Nordberg Karlsson

**Affiliations:** 1 Division of Biotechnology, Department of Chemistry, Lund University, Naturvetarvägen 14, 22100 Lund, Sweden; 2 Matis Ohf, Vinlandsleid 12, 113 Reykjavik, Iceland; 3 Department of Biology, School of Engineering and Natural Sciences, University of Iceland, Sturlugata 7, 102 Reykjavik, Iceland

**Correction to: Microbial Cell Factories (2022) 21:220** **https://doi.org/10.1186/s12934-022-01946-7**

In the original publication of the article [1], Table 6 has been processed which was already available in the additional file. Hence, the Table 6 has been removed and the Table 7 has been renumbered to Table 6. The original article has been corrected.
