# Supplementary material for: Medium development and production of carotenoids and exopolysaccharides by the extremophile Rhodothermus marinus DSM16675 in glucose-based defined media
Source: Microb Cell Fact. 2022 Oct 23;21:220. doi: 10.1186/s12934-022-01946-7 (PMC9590192; doi:10.1186/s12934-022-01946-7)
Supplement: Supplementary file 1 — Additional file 1: Table S1. Screening experiments of four macromolecules (CaSO4, MgCl2, PO43−, and NH4Cl) with one factor at a time screening strategy (A, B, C, D, E) and combination of high levels of two factors at a time and keeping others at the concentrations in the original DMB medium (BC, BD, BE, CD, CE, DE). Table S2. The growth profile of R. marinus DSM 16675 in DRM medium with different concentration of glucose as sole carbon source. Table S3. The preparation condition of DRM. (A) Preparation condition for stock solutions (B) The preparation condition for DRM with final concentration in the medium. The pH is adjusted to 7.2 by NaOH (6 M) after addition of the remaining water. Table S4. The relative monosaccharide composition (molar ratio) of the purified EPSs produced by R. marinus DSM 16675 in RDM. The amount of glucose is set as 1. Table S5. Uptake and secretion rates of selected metabolites. In vivo rates were determined from experimental data in Fig. 7. In silico rates were obtained by simulating maximum growth rate in a genome-scale metabolic model of R. marinus. Figure S1. The monosaccharides chromatograms of the purified EPSs produced by R. marinus DSM 16675 in DRM with two unidentified peaks. Figure S2. FT-RI spectrum of the purified exopolysaccharide from R. marinus DSM 16675 grown in DRM. [file 12934_2022_1946_MOESM1_ESM.docx]

**Table S1:** Screening experiments of four macromolecules (CaSO_4_, MgCl_2_, PO_4_^3-^, and NH_4_Cl) with one factor at a time screening strategy (A, B, C, D, E) and combination of high levels of two factors at a time and keeping others at the concentrations in the original DMB medium (BC, BD, BE, CD, CE, DE).

| **Medium** | **NH_4_Cl (g/L)** | **Na_2_HPO_4_ (g/L)** | **KH_2_PO_4_ (g/L)** | **MgCl_2_ × 6H_2_O (g/L)** | **CaSO_4_ × 2H_2_O (g/L)** |
| --- | --- | --- | --- | --- | --- |
| A (DMB) | 0.535 | 0.28 | 0.27 | 2 | 0.4 |
| B | 1.6 | 0.28 | 0.27 | 2 | 0.4 |
| C | 0.535 | 1.41 | 1.36 | 2 | 0.4 |
| D | 0.535 | 0.28 | 0.27 | 4 | 0.4 |
| E | 0.535 | 0.28 | 0.27 | 2 | 2 |
| BC | 1.6 | 1.41 | 1.36 | 2 | 0.4 |
| BD | 1.6 | 0.28 | 0.27 | 4 | 0.4 |
| BE | 1.6 | 0.28 | 0.27 | 2 | 2 |
| CD | 0.535 | 1.41 | 1.36 | 4 | 0.4 |
| CE | 0.535 | 1.41 | 1.36 | 2 | 2 |
| DE | 0.535 | 0.28 | 0.27 | 4 | 2 |

**Table S2.** The growth profile of *R. marinus* DSM 16675 in DRM medium with different concentration of glucose as sole carbon source

**Table S3:** The preparation condition of DRM. (A) Preparation condition for stock solutions (B) The preparation condition for DRM with final concentration in the medium. The pH is adjusted to 7.2 by NaOH (6 M) after addition of the remaining water.

**Table S4.** The relative monosaccharide composition (molar ratio) of the purified EPSs produced by *R. marinus* DSM 16675 in RDM. The amount of glucose is set as 1.

| **Time (h)** | **CDW (g)** | **ln(CDW)** | **Sulfate (mM)** | **Sulfate (mM)** | **Phosphate (mM)** | **Phosphate (mM)** | **Ammonium (mM)** |
| --- | --- | --- | --- | --- | --- | --- | --- |
| 0 | 0.2 | -1.609 | 0.2 | 2 | 4 | 20 | 10 |
| 18 | 2.85 | 1.047 | 0 | 0 | 0 | 0 | 0 |
| *In vivo* rates (mmol/CDW/h) | | | -0.0111 | -0.1114 | -0.2228 | -1.1139 | -0.5570 |
| *In silico* rates (mmol/CDW/h) | | | -0.047 | | -0.20 | | -1.98 |

**Table S5:** Uptake and secretion rates of selected metabolites. *In vivo* rates were determined from experimental data in **Fig. 7**. *In silico* rates were obtained by simulating maximum growth rate in a genome-scale metabolic model of *R. marinus*.

Figure S1. The monosaccharides chromatograms of the purified EPSs produced by *R. marinus* DSM 16675 in DRM with two unidentified peaks.

**Figure S2.** FT-RI spectrum of the purified exopolysaccharide from *R. marinus* DSM 16675 grown in DRM.
